# Editorial: Integrating sensors and artificial intelligence for objective pain detection and quantification: unveiling new possibilities

**DOI:** 10.3389/fpain.2025.1654743

**Published:** 2025-07-09

**Authors:** Youngsun Kong, Raul Fernandez Rojas, Hugo F. Posada-Quintero

**Affiliations:** ^1^ Department of Biomedical Engineering, University of Connecticut, Storrs, CT, United States; ^2^ Human-Centred Technology Research Centre, Faculty of Science and Technology, University of Canberra, Canberra, ACT, Australia

**Keywords:** pain detection, pain quantification, sensor technologies, artificial intelligence, clinical applications, pain management

**Editorial on the Research Topic**
Integrating sensors and artificial intelligence for objective pain detection and quantification: unveiling new possibilities

Pain remains one of the most challenging aspects of modern medicine due to its inherently subjective and multifaceted nature. The development of accurate and objective methods for pain assessment holds significant potential to improve diagnosis and treatment across a wide range of medical conditions. Motivated by growing interest in this field, we launched this Research Topic to highlight recent advancements in objective pain quantification, particularly through the use of sensor technologies and artificial intelligence (AI).

This Research Topic features four original research articles that collectively showcase innovative approaches and emerging trends in the measurement and analysis of pain.

The first article, by Winslow et al., introduces a logistic regression-based algorithm using respiratory and heart rate variability features extracted from electrocardiography (ECG) signals to objectively detect acute pain induced by cold pressor testing. Their model achieved an F1 score of 81.9% for laboratory/clinical settings and 79.4% for field/ambulatory settings, based on data from 41 participants using the leave-one-subject-out cross-validation (LOSO-CV) method—a robust validation approach for machine learning approaches. Their work demonstrates the feasibility of binary pain detection using ECG data, which can be readily collected from both wearable and clinical devices.

The second article, by Gkikas et al., advances the field through a multimodal approach integrating ECG-derived heart rate and facial video data. Their transformer-based framework includes four key modules: a spatial module for extracting embeddings from videos, a heart rate encoder to map signals into higher-dimensional space, AugmNet to create latent-space augmentations, and a temporal module for final classification. Their model was evaluated on the publicly available BioVid dataset, which includes heat pain stimuli from 87 healthy participants, using the LOSO-CV method. It achieved 82.74% accuracy for binary and 39.77% for multi-level pain classification tasks.

Labeling long-term pain data remains one of the most significant challenges in developing pain detection systems, particularly for chronic pain assessment. To address this, Ricken et al. developed a pseudo-labeling algorithm that transfers knowledge from short-term to long-term pain domains by iteratively generating high-confidence labels. Their method enables robust classification across multiple sensor modalities (e.g., ECG, electromyography, electrodermal activity) using the public X-ITE pain database, which includes thermal and electrical pain stimuli from 124 healthy subjects. Using random forest classifiers with the LOSO-CV method, their algorithm improved classification performance by 2.4%–2.8%, up to 80.4% accuracy compared to baseline models.

While these methods demonstrate the technical feasibility of objective pain quantification, questions remain regarding their clinical utility. Fu et al. addressed this by introducing the Pain Intervention and Digital Research (Pain-IDR) Program, which integrates outpatient clinical care with digital health research for older adults with chronic pain. Their pilot program demonstrated the feasibility of remote, active (e.g., surveys) and passive (e.g., smartphone sensor data) monitoring via a smartphone application. Among 77 participants (mean age: 55.52), 38 completed the full 6-month study. The program achieved an active data collection rate of 51% and a passive data rate of 78%.

This Research Topic successfully highlights modern technologies leveraging sensors and AI for objective pain assessment, with promising potential for clinical integration. We hope readers find these contributions insightful and impactful for future research and practice in digital health and pain quantification.

